# Quality of life and psychological distress during cancer: a prospective observational study involving young breast cancer female patients

**DOI:** 10.1186/s12885-020-07272-8

**Published:** 2020-08-13

**Authors:** Barbara Muzzatti, Francesca Bomben, Cristiana Flaiban, Marika Piccinin, Maria Antonietta Annunziata

**Affiliations:** Centro di Riferimento Oncologico di Aviano (CRO), IRCCS, Via F. Gallini, 2, 33081 Aviano, PN Italy

**Keywords:** Breast cancer, 18–45 year-old, Females, Quality of life, Psychological distress

## Abstract

**Background:**

Despite the possible traumatic significance of cancer and of the incidence, prevalence, and survival of young women with breast cancer, these patients are underrepresented in multidimensional research. In the present survey, QoL and psychological distress were studied in a sample of young female breast cancer patients during the first year of their disease. More in detail, the study was firstly aimed to assess if QoL of 18–45 years old female breast cancer patients was different from QoL of women from the general population and if it changed over time. Secondly, it described the psychological distress and its change over time. Finally, it assessed if QoL registered 1 year post-surgery may be explained by QoL and/or psychological distress registered during the hospitalization.

**Methods:**

One hundred six, consecutive 18–45 years old, female primary breast cancer patients undergoing anticancer surgery filled out the Short Form 36 Health Survey Questionnaire, the Hospital Anxiety and Depression Scale and a socio-demographic and clinical form during hospitalization to receive surgery (T0), and again at 12 months post-surgery (T1).

**Results:**

At T0, participants showed a better physical functioning (*p = 0*.001) than the female normative sample, whereas their mental functioning was worse (*p < 0*.001). In this time, mental functioning within our sample was weaker than physical functioning (*p < 0*.001). Over time, physical functioning decreased (*p < 0*.001), whereas mental functioning increased (*p < 0*.001). Both at T0 and T1, anxiety was higher than depression (*p < 0*.05). Both distress dimensions decreased over time (*p < 0*.001). Nevertheless, at T0 the 25.5 and 26.4% of the sample were respectively possible and probable cases of anxiety, whereas the 17.9 and 9.4% were possible and probable cases of depression. At T1, the percentages were 17.9 and 18.9% for anxiety, and 8.5 and 6.6% for depression. In both considered times, a better QoL corresponded to less psychological distress. However, QoL and psychological distress assessed at T0 did not predict the QoL at T1.

**Conclusions:**

This study documented as QoL and psychological distress may change during the first year after surgery for a primary breast cancer in young women; therefore, they should be monitored over time to detect and treat women with alarming levels on them.

## Background

Cancer is a para-normative event that interferes with a person’s life trajectory, projects, and plans in the short, medium, and sometime also the long term [1]. Since it threats a person’s safety and since it often requires demanding treatments, it is included among the possible traumas that can occur during life [2]. The impact of cancer in terms of decrements in psychological well-being and quality of life is well documented in literature for different cancer types and for different steps of the disease trajectory. In particular, females [3, 4] and younger people [4–7] seem to be more vulnerable than their counterparts.

According to the psychologist psychoanalyst Erik Erikson, Generativity, in its biological meaning of parenthood as well as in its broader meaning of self-realization (i.e. the way in which each individual expresses him/herself, finds his/her place in the world, gives his/her personal contribution to the society) is the developmental task of adulthood [8]. If a person were impaired to pursue it, he/she might fall into stagnation [8]. Getting a cancer during adulthood may interfere with this task both directly (infertility, stall in working career) and indirectly (psychological distress, fear of recurrence, body image problems).

Breast cancer is the most frequently diagnosed malignancy in Italian women, accounting for 29% of all newly diagnosed cancers in women (41% between 0 and 49 years); it is estimated that one every eight women develops a breast cancer during her life span [9]. In 2018, in Italy, 52,800 women had breast cancer. The risk of getting breast cancer varies according to age, and it is 2.4% up to 49 years of age [9]. The 5-year survival rate for breast cancer was 87% in Italy, and it rose to 91% for women aged 15–44 years. Breast cancer caused death in 29% of cases among women under 50 years of age.

The possible traumatic significance of cancer *per sè* together with the incidence, prevalence, and survival of breast cancer justify the investigation of quality of life (QoL) and psychological distress in young female patients. However, according to the ESO-ESMO 3rd international consensus guidelines for breast cancer in young women [10], young breast cancer patients are underrepresented in multidimensional research, despite the fact that they are often affected by more aggressive cancer types, have less favorable outcomes, are vulnerable to psychosocial distress at diagnosis and later during their disease trajectory.

Avis and colleagues [11] demonstrated that the general QoL of a sample of breast cancer patients aged 50 years or less was worse than QoL of non patients. A recent review on QoL in young breast cancer women [12] reported that fatigue, pain in the breast, and hand problems with lymphedema were the most frequently mentioned physical effects, followed by irregular and painful menses, and sexual difficulties. In addition, it reported that many young women had depressive symptoms (depressed mood, helplessness, hopelessness, psychomotor retardation, and disorders of appetite and/or sleep), concerns about the health and self-image, and worries about motherhood and children-bearing. Finally, according to the same review, isolation problems and feeling different from other women of similar age were experienced by breast cancer female patients together with barriers in their working career including discrimination in labor supply and/or layoff.

Despite these data, further information on both QoL and psychological distress, as well as on their trajectory during cancer, in young female breast cancer patients, are necessary to organize interventions tailored on their actual needs or aimed to prevent later difficulties. This study was designed to provide further data in this field. More in detail, the study was firstly aimed to assess if QoL of 18–45 years old female breast cancer patients was different from the QoL of women from the general population and if it changed over time. Secondly, it described the psychological distress and its changes over time. Finally, it assessed whether QoL registered 1 year post-surgery may be explained by QoL and/or psychological distress registered during hospital stay. For this study a large age range, from 18 to 45 years old, was chosen. The choice was motivated by the already mentioned underrepresentation of young breast cancer female patients in multidimensional research [10]; the clinical specificities of this period of life, related to cancer occurrence and the impact of cancer treatments [8, 11, 12]; and especially epidemiological data showing a 50% increase in breast cancer incidence in Italian women aged 39–44 years compared to 20–39 years-old [13].

## Method

### Participants

All participants were 18–45 years old female consecutive patients undergoing anticancer surgery for a primary breast cancer in the same cancer institute in the North-east of Italy. Further study inclusion criteria were the ability to understand the Italian language, having signed the informed consent form, and having provided complete data in both study assessments. A diagnosis different from breast cancer or presence of metastases were exclusion criteria for the study.

### Procedure and materials

The study was both observational and prospective, and it consisted of two subsequent QoL and psychological distress assessments. The first occurred during participants’ hospital stay, after breast cancer surgery and before discharge (T0); the second occurred 12 months after the first one (T1).

Eligible participants were identified by consultation of clinical records. A researcher approached each potential candidate, illustrated the study in its aims and procedures, obtained a written consent to participate, and delivered materials for the first administration. Participants filled out the questionnaires alone during their hospital stay.

For the second assessment, 1 year later a researcher reached by phone each participant to remind them about the study and that they had given permission to receive study materials at home. Participants filled out the questionnaires and returned them by mail (a pre-paid envelope was provided together with the study booklet) within 3 weeks.

The Institute Independent Ethics Committee gave its clearance to the study.

In both assessments, participants were required to compile the Short Form 36 Health Survey Questionnaire (SF-36, [14]), and the Hospital Anxiety and Depression scale (HADS [15];). A form to collect socio-demographic and clinical data was also administered at T0.

The SF-36 is a multidimensional QoL measure, consisting of 36 items and eight different QoL indices: Physical Functioning, Role-Physical Limitation, Bodily Pain, General Health, Vitality, Social Functioning, Role-Emotional Limitation, Mental Health. In each index, higher scores indicate better functioning in that domain. Physical Functioning, Role-Physical Limitation, Bodily Pain and General Health may be merged in a comprehensive index for physical functioning (the Physical Component Summary [PCS]), as well as Vitality, Social Functioning, Role-Emotional Limitation and Mental Health may compose a comprehensive index of mental functioning (the Mental Component Summary [MCS]). For both PCS and MCS raw scores are converted in t-scores (with mean = 50 and standard deviation = 10). Apolone and colleagues [16] validated the Italian version of SF-36 and provided reference data. A generic QoL measure (i.e., SF-36), rather than a cancer-specific (or breast cancer-specific) measure, was used because the items of this kind of questionnaires seemed to be more suitable for 1-year post-surgery participants (in fact in generic measures items about cancer and/or anticancer treatments like nausea, vomiting, diarrhea, hear loss, etc. are not present). Furthermore, SF-36 was chosen for its good psychometric properties as well as for the availability of reference data for Italy.

The HADS is a self-report scale assessing psychological distress in its main components of anxious and depressive states. It is made up of 14 Items merged in two factors: Anxiety and Depression [17]. In each HADS factor, higher scores correspond to higher anxious or depressive state. Zigmond and Snaith [15] offered instruction for HADS interpretation.

Socio-demographic and clinical data were self-reported in the first assessment; information on age, marital status, education, occupational status, and undergoing cancer treatment were collected. Information on anticancer treatments received during the first year since breast surgery were obtained by consulting clinical files.

**Fig. 1:**
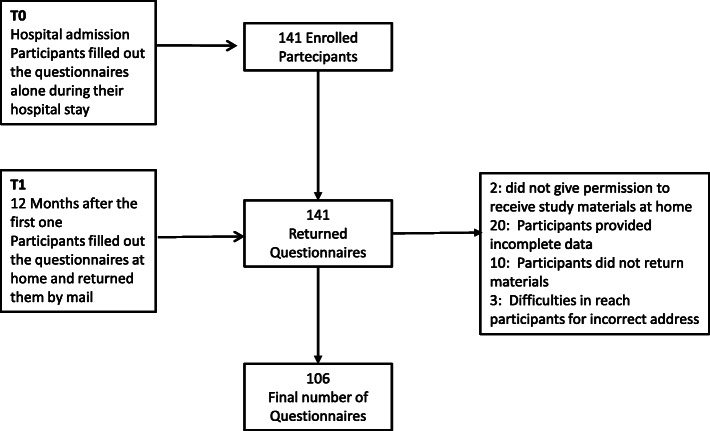
Study flow diagram

Study materials were delivered to 141 participants of whom, 106 (75.2%) completed both questionnaires and thus were included in the study (Fig. 1).

### Statistical analysis

Descriptive statistics (mean, standard deviation, and frequencies) were calculated for the two SF-36 comprehensive indices. One-sample t-tests were performed to compare sample data with norms (i.e., 1032 Italian females from the general population [16]) in both T0 and T1. Paired-samples t-tests were performed to compare both physical and mental functioning within time. Paired-sample t-tests were also performed to compare physical and mental functioning within each assessment time.

For each of the eight factors of Sf-36, descriptive statistics were provided, and the one-sample t-test was performed to compare the present sample data with the norms in T0 and T1. In addition, paired-sample t-tests were performed to compare each SF-36 factor scores within times.

Descriptive statistics and frequencies were provided for both psychological distress dimensions (i.e. anxiety and depression). Paired sample t-tests were carried out to compare both anxiety and depression within time as well as to compare anxiety and depression in the same assessing time.

Pearson correlations were calculated to assess the associations between QoL (comprehensive indices) and psychological distress in T0 and T1. In addition, two-sample independent t-tests were performed to compare PCS and MCS scores (for each time) between anxiety (or depression) non cases vs. possible/probable cases.

To assess whether T1 QoL could had been predicted by QoL and/or psychological distress assessed during T0, for both QoL comprehensive indices, a multiple-linear regression model was calculated using psychological distress (anxiety and depression) and QoL comprehensive indices assessed at T0 as predictors.

In all analyses, *p* < 0.05 (2-tailed) was used for statistical significance. Regarding clinical and social significance, on a scale of 0–100, a difference of 5 units was considered significant (CMD) [16]. All statistical analyses were carried out using SPSS software, Version 20 (SPSS Inc., Chicago, Ill.).

### Sample size

The Italian SF-36 manual [16] establishes that the necessary sample to identify a five-point difference between the average of a group and reference norm is 32 for both comprehensive indices (PCS, MCS), considering alpha =0.05, power = 80% and applying a two-tail t-test.

## Results

### Sample characteristics

The final sample consisted of 106 participants; the median age was 40.5 years (range: 25–45 years), and 39 (36.8%) were females aged < 40 year-old. Eighty-eight (83.8%) participants reported a post-compulsory education (i.e., more than 8 years of schooling); 78 (73.6%) reported to be in a stable relationship (i.e., being married or cohabiting); and 84 (79.2%) reported having a paying job. 53 (50%) and 53 (50%) had received quadrantectomy and mastectomy, respectively. Thirty women (56.6%), receiving quadrantectomy, and 26 women (49.1%), receiving mastectomy, underwent axillary lymph node dissection. During breast surgery, 10 (9.4%) women also underwent to breast reconstruction, whereas 4 patients (3.8%) received intraoperative radiotherapy. After surgery, 62 women (58.5%) received adjuvant chemotherapy (for 6–24 weeks), 44 received adjuvant radiotherapy, 81 (76.4%) hormonotherapy, and 16 (15.1%) monoclonal antibodies.

### Physical and mental comprehensive functioning during cancer

Table 1 reports comprehensive indices for physical and mental functioning (i.e. PCS and MCS) registered at T0 and T1. At T0, participants showed a better physical functioning (*p = 0*.001) than the female normative sample, whereas their mental functioning was worse (*p < 0*.001; CMD). During this time, mental functioning was weaker than physical functioning (*p < 0*.001; CMD).

**Table 1:** Physical and mental comprehensive functioning (*N* = 106) during hospital stay for breast surgery and 1 year later: means and (standard deviations)

	Hospital stay	1-year later	*P*	Norms [16]
Physical functioning	51.8^+^ (9.5)	47.1 (9.0)	0.000	48.7
Mental functioning	38.2^#^ (11.0)	45.5 (11.0)	0.000	44.8
*p*	0.000	0.218	–	–

^+^*p* < 0.01; comparison with norms. ^#^*p* < 0.001 comparisons with norms

At T1, neither differences from normative data nor within subjects were statistically significant. Over time, physical functioning decreased (*p < 0*.001), whereas mental functioning increased (*p < 0*.001; CMD).

At T0, 11.3 and 1.9% of the sample displayed respectively a score less than one and two standard deviation from the normative mean score of 50 in physical functioning; in mental functioning, the percentages reached to 34.9 and 21.7% respectively. At T1, 17.9 and 2.8% of the sample displayed respectively a score less than one and two standard deviation from the normative mean score of 50 in physical functioning; in mental functioning, the percentages reached to 14.2 and 11.3% respectively.

### Detailed QoL domains during cancer

Table 2 reports the eight QoL domains recorded by SF-36 at T0 and T1. At T0, participants showed less bodily pain (*p = 0*.001; CMD), a poorer social functioning (*p < 0*.001; CMD), more limitations due to emotional causes (*p < 0*.001; CMD), and a poorer mental health (*p = 0*.001; CMD) than the female normative sample. At T1, in comparison with the norms, the same sample displayed a better physical functioning (*p = 0*.026) and a better mental health (*p < 0*.001; CMD), but it displayed more limitations due to physical causes (*p < 0*.001; CMD).

**Table 2:** QoL domains (*N* = 106) during the hospital stay for breast surgery and 1 year later: means and (standard deviations)

	Hospital stay	1-year later	***P***^**a**^	Norms^**b**^ [16]
Physical Functioning	81.3 (25.4)	84.3 (15.2)	0.216	81.02
Role-Physical Limitation	69.8 (38.7)	58.3 (41.8)	0.028	74.13
Bodily Pain	76.8 (23.1)	68.6 (254)	0.008	69.01
General health	65.4 (20.5)	63.3 (21.3)	0.312	63.19
Vitality	58.5 (17.7)	56.2 (19.2)	0.278	57.98
Social Functioning	59.6 (25.1)	70.6 (23.2)	0.000	73.89
Role-Emotional Limitation	48.7 (38.5)	68.2 (38.6)	0.000	71.78
Mental Health	56.0 (19.3)	67.8 (17.3)	0.000	62.48

^a^Values from the comparisons between T0 and t1. ^b^1032 Italian females from the general population [16]

In comparison with T0, at T1 participants displayed more limitations due to physical causes (*p = 0*.028; CMD), less pain (*p = 0*.008; CMD), a better social functioning (*p < 0*.001; CMD), less limitations due to emotional causes (*p* < 0.001; CMD), and a better mental health (*p* < 0.001; CMD).

### Psychological distress during cancer

Table 3 shows the psychological distress recorded by HADS at T0 and T1. At both times, anxiety was higher than depression (*p = 0*.002; *p = 0*.012). Furthermore, both of them decreased over time (*p < 0*.001).

**Table 3:** Psychological distress (*N* = 106) during the hospital stay for breast surgery and 1 year later: means and (standard deviations)

	Hospital stay	1-year later	***P***
Anxiety	8.1 (4.0)	6.9 (4.1)	0.002
Depression	5.4 (3.6)	4.4 (3.6)	0.012
*P*	0.000	0.000	–

Nevertheless, at T0 the 25.5 and 26.4% of the sample were respectively possible and probable cases of anxiety, whereas the 17.9 and 9.4% were possible and probable cases of depression. At T1, the percentages were 17.9 and 18.9% for anxiety, and 8.5 and 6.6% for depression.

### Associations between QoL comprehensive indices and psychological distress

At T0, an inverse correlation between PCS and depression was obtained (*R* = − 0.375; *p < 0*.001), whereas MCS was inversely correlated with both anxiety (*R* = −0.547; *p* < 0.001) and depression (*R* = − 0.560; *p < 0*.001). The 55 participants who were possible or probable cases of anxiety displayed worse scores in MCS (M = 33.1 vs. 43.7; *p < 0*.001; CMD) than non cases (*N* = 51). The 29 participants who were possible or probable cases of depression displayed worse scores in both PCS (M = 45.8 vs. 54.1; *p < 0*.001; CMD) and MCS (M = 30.5 vs. 41.1; *p < 0*.001; CMD) than non cases (*N* = 77).

At T1, PCS was inversely correlated with anxiety (*R* = -0.281; *p = 0*.003) and depression (*R* = -0.331; *p = 0*.001) as well as MCS (*R* = -0.761; *p < 0*.001) (*R* = -0.711; *p < 0*.001). The 39 participants who were possible or probable cases of anxiety displayed worse scores in both PCS (M = 44.7 vs. 48.5; *p = 0*.040) and MCS (M = 36.0 vs. 51.0; *p < 0*.001; CMD) than non cases (*N* = 67). The 16 participants who were possible or probable cases of depression displayed worse scores in both PCS (M = 42.2 vs. 48; *p = 0*.017) and MCS (M = 31.1 vs. 48.1; *p < 0*.001; CMD) than non cases (*N* = 90).

The estimation model for the association of PCS at T1 with QoL and psychological distress assessed at T0 explained 6.7% of the total variance (*p* = 0.131). In this model, T1 PCS was predicted by neither of the considered regressors. However, when MCS, depression and anxiety scores assessed at T1 were added to the model, the percentage of the explained total variance reached 24.8% (*p < 0*.001). According to this second model, T1 PCS was associated with T0 PCS (*p = 0*.023), whereas its association was negative with concurrent MCS (*p < 0*.001), anxiety (*p = 0*.046), and depression (*p = 0*.002) (Table 4).

**Table 4:** Standardized Beta coefficient estimates of physical and mental comprehensive functioning one-year after breast cancer surgery

Regressor	Physical *Functioning*		Mental Functioning	
	Beta	*p*	Beta	*P*
Physical functioning during hospital stay	0.272	0.023	0.172	0.026
Mental functioning during hospital stay	0.121	0.371	0.075	0.386
Anxiety during hospital stay	−0.008	0.952	0.047	0.592
Depression during hospital stay	0.171	0.270	0.122	0.225
Physical functioning 1-year later	–	–	−0.231	< 0.001
Mental functioning 1-year later	−0.555	< 0.001	–	–
Anxiety 1-year later	−0.339	0.046	−0.560	< 0.001
Depression 1-year later	−0.465	0.002	−0.395	< 0.00
	*R* = 0.498; *R*^2^ = 0.248; F(7, 105) = 4.614, *p* < 0.001; computed by means of a multivariate linear model		*R* = 0.829; *R*^2^ = 0.687; F(7, 105) = 30.768, *p* < 0.001; computed by means of a multivariate linear model	

The estimation model for the association of T1 MCS with QoL and psychological distress assessed at T0 explained 19.4% of the total variance (*p* < 0.001). In this model, T1 MCS was inversely associated with anxiety (*p = 0*.020). However, when PCS, depression and anxiety scores concurrently assessed were added to the model, the percentage of the explained total variance reached 68.7% (*p < 0*.001). According to this second model, T1 MCS was associated with T0 PCS (*p = 0*.026) and it was negatively associated with concurrent PCS, anxiety, and depression (*p < 0*.001) (Table 4).

## Discussion

This prospective study involved a sample of 25–45 years-old female breast cancer consecutive patients, of whom 63% were aged 40–45 years. All participants were in the stage named “generativity versus stagnation” [8]; consequently, despite the wide age range, all of them might face analogous developmental goals. We assessed both QoL and psychological distress twice: after surgery and one-year later. Generally, adjuvant therapy starts about 1 month after breast surgery and lasts about 3–6 months [18]. Quadrantectomy is often followed by radiotherapy [18]. QoL and emotional effects of both diagnosis and treatments, free from the emotional impact provoked by negative effects of active treatments (such as asthenia or fatigue) arise (consequently may be detected) after 1 year from the diagnosis [19]. This happens because during treatments, patients are mentally occupied by physical issues (such as management of side effects, saving their own life) and this mental status endures until an acceptable physical recovery had been reached. Consequently, the chosen period might allow to capture the consequences of a full awareness of what had happened (the diagnosis, treatments) and its implications for the future, on both QoL and psychological functioning.

In this study, both QoL comprehensive indices (i.e. physical and mental functioning summary scores) assessed 1 year after breast cancer surgery were comparable to those of the norms. More in detail, the assessed sample displayed better physical functioning and mental health together with more limitations due to physical causes than the norms, whereas no differences were found in the other five QoL detailed domains. In SF-36, Physical functioning assesses the presence and interference of impairments in basic daily life activities, such as toiletry, having a walk, lifting weights; Mental health corresponds to an enduring state of calmness and serenity; Limitations due to physical health comprise reduction in the time spent working and/or having other role-related activities, difficulties and limitations in doing work and/or other activities [14]. Consequently, we can state that 1 year post-surgery the QoL of the enrolled sample was normal except for the presence of difficulties related to work and/or other activities defining a person’s role. However, we must also remember that about 20 and 25% of the sample revealed a poor functioning in physical and mental comprehensive QoL components respectively. The datum dealing with the presence of difficulties related to work and/or other activities defining a person’s role finds an explanation in a previous qualitative study investigating work perception in breast cancer survivors aged 40 years or less [20]. In fact, this study revealed how experiencing breast cancer was viewed by participants as contributing to an increased desire for work to provide a sense of meaning. In addition, breast cancer was associated with loss of control over career success and work choices; treatment side effects were described as interfering with work self-efficacy and skills; and interpersonal difficulties connecting within and outside of work were reported. Difficulties connected to body image and sexuality, management of career and finances, management of children and everyday life had been also described for young breast cancer women and their spouses by Duprez and colleagues [21].

During the hospital stay to receive breast surgery, the QoL profile was more composite. The physical comprehensive summary indicated a better QoL physical component than the norms, whereas the mental QoL component was worse. More in details, participants displayed less bodily pain (or limitations due to it), a poorer social functioning (i.e., limitations in personal social life due to physical or emotional problems), more limitations due to emotional causes (i.e., limitations in work and/or in other role-related activities due to emotional problems) and a poorer mental health (i.e., a pervasive state of anxiety and nervousness) than the female normative sample. Moreover, more than 13 and 56% of the sample displayed a lacking comprehensive physical and mental QoL functioning. Consequently, according to current data, the hospital admission to receive breast surgery corresponded to a poor QoL, especially in its mental component. This datum corroborated the literature describing poor QoL in patients within a month from breast cancer diagnosis [22].

Furthermore, it corroborated previous research [23] in which anxiety was found higher at diagnosis than later, in young female breast cancer patients.

During cancer, QoL changed in its comprehensive components as well as in several detailed domains. In fact, one-year post breast surgery, social functioning, mental health and limitations due to emotional causes improved, whereas limitations due to physical causes increased. This pattern of results is quite different from that described by Montazeri and colleagues [24] who assessed QoL in a sample of 99 breast cancer patients at diagnosis and at 18 months later. According to Montazeri et al. study, physical functioning improved whereas both emotional functioning and general QoL decreased over time.

Over time, also the psychological distress (in its two components of anxiety and depression) decreased; however, 1 year after breast surgery approximately one every five patients displayed a probable anxiety, a proportion that grew to one every three if both possible and probable cases of anxiety were considered. In both assessments, anxiety was stronger than depression. Conversely, Costanzo and colleagues [5] assessed psychological distress in 99 women under treatment for breast cancer and 3 weeks and 3 months after the end of treatment, and no reduction in distress components was found. In both considered times, a better QoL corresponded to less psychological distress. However, neither QoL nor psychological distress at first assessment predicted the QoL at the second one. The association between the psychological distress and QoL is well established in the literature and supports the management of psychological distress to improve the patient’s well-being and QoL. The poor association between the two subsequent QoL assessments could sound non surprising too, if we take into consideration the situational nature of QoL. However, the clinical implication of this datum is relevant, since it implies the need for repeated QoL assessments during the disease trajectory to timely detect its lack. In other words, present data suggested that a good psychological adjustment (i.e., no anxiety or depression, QoL comparable to QoL of the female general population) to a breast cancer diagnosis and surgery did not imply unlikelihood of late decrements in QoL.

The main limitation of the present study rests on the specific sample enrolled (i.e. 18–45-year-old cancer patients undergoing breast surgery) and procedure (only 2 subsequent assessments), which impair generalizations. Furthermore, lack of information on the cancer stage and on the treatments (especially on the psychological ones) to which the patients were subjected between T0 and T1 prevents to clarify any confounders or factors that may have affected the results after 1 year. Finally, norms for the Italian version of SF-36 were dated back to 1997; despite being the only norms available, it is possible that they cannot completely capture actual QoL of Italian population. At the same time, the sample homogeneity and its being composed by consecutive patients, together with the study power and the tools used (i.e. validated and standardized questionnaires) are a strength. Future research should expand these findings to other steps of the disease trajectory (for instance, a third assessment, few months to 1 year post-treatment would be interesting because many dimensions of QoL as well as the intensity of distress can further evolve during the post-treatment period) as well as to other kind of patients, i.e., different age and/or with different cancer diagnosis. Moreover, an in-depth investigation of the impact of cancer in working and daily life will be useful. For instance, in the present study, 84 out of 106 participants declared to have a job; of them, 65 stated to be employees, and 19 reported to be a businesswoman, a freelance, or a handicraft worker. We did not collect data on possible changes in participants’occupational status (including reduction in working time) during the first year of disease trajectory, but this topic surely requires more attention. The relationship between the type of work (e.g., permanent or non-permanent) and QoL and well-being during cancer should also be studied. This study focused on QoL and psychological distress; nevertheless, other psychological dimensions, such fear of a disease recurrence or body image, are relevant in describing illness in this kind of patients and, consequently, should be investigated.

## Conclusions

This study documented as QoL and psychological distress may change during the first year after surgery for a primary breast cancer in young women. In particular, it showed that mental functioning after breast surgery was lower than that of female general population; mental functioning increased over time and psychological distress decreased, even though a non negligible proportion of the sample displayed psychological distress one-year post-treatment; moreover, both QoL and psychological distress, registered during hospital stay for breast surgery, did not predict QoL registered one-year post breast surgery. Our findings suggested that this kind of patients should be monitored over time, in order to detect and treat those who display alarming levels in these dimensions, despite their QoL and distress levels in the first steps of the disease trajectory.

## Data Availability

The dataset used and analyzed during the current study is available from the corresponding author on reasonable request.

## Abbreviations

CMD: Clinically meaningful difference
HADS: Hospital Anxiety and Depression Scale
MCS: Mental Component Summary
PCS: Physical Component Summary
QoL: Quality of life
SF-36: Short Form 36 Health Survey Questionnaire
